# NFATc3 controls tumour growth by regulating proliferation and migration of human astroglioma cells

**DOI:** 10.1038/s41598-019-45731-w

**Published:** 2019-06-27

**Authors:** Katia Urso, Andrés Fernández, Patricia Velasco, Javier Cotrina, Belén de Andrés, Pilar Sánchez-Gómez, Aurelio Hernández-Laín, Sonsoles Hortelano, Juan Miguel Redondo, Eva Cano

**Affiliations:** 1 Chronic Disease Programme, Madrid, Spain; 2 Centro Nacional de Microbiología, Madrid, Spain; 3 Instituto de Enfermedades Raras, Instituto de Salud Carlos III, Madrid, Spain; 4 Department of Vascular Biology and Inflammation, Centro Nacional de Investigaciones Cardiovasculares (CNIC), Madrid, Spain; 5 Hospital Doce de Octubre, Madrid, Spain

**Keywords:** Calcium signalling, CNS cancer, CNS cancer

## Abstract

Calcium/Calcineurin/Nuclear Factor of Activated T cells (Ca/CN/NFAT) signalling pathway is the main calcium (Ca^2+^) dependent signalling pathway involved in the homeostasis of brain tissue. Here, we study the presence of NFATc members in human glioma by using U251 cells and a collection of primary human glioblastoma (hGB) cell lines. We show that NFATc3 member is the predominant member. Furthermore, by using constitutive active NFATc3 mutant and shRNA lentiviral vectors to achieve specific silencing of this NFATc member, we describe cytokines and molecules regulated by this pathway which are required for the normal biology of cancer cells. Implanting U251 in an orthotopic intracranial assay, we show that specific NFATc3 silencing has a role in tumour growth. In addition NFATc3 knock-down affects both the proliferation and migration capacities of glioma cells *in vitro*. Our data open the possibility of NFATc3 as a target for the treatment of glioma.

## Introduction

Gliomas are among the most severe forms of cancer. Gliomas originate from glia cells (astrocytes, oligodendrocytes, ependymal cells) or cancer stem cells, and are classified by the World Health Organization (WHO) into four grades based on malignancy (I to IV). The most common of these fatal tumours is grade IV astrocytoma or glioblastoma (GBM) and the standard treatment is surgical resection followed by radiation and chemotherapy, which does not dramatically improve clinical outcome.

Calcium (Ca^2+^) dependent signalling pathways are pivotal in the homeostasis of brain tissue. A key element of the cellular response to Ca^2+^ signals is the action of calcineurin (CN), a Ca^2+^- and calmodulin-dependent phosphatase firstly discovered in brain tissue, where is highly abundant^1^ and described later in many other tissues (reviewed in^2,3^). CN activation regulates the activation of the Nuclear Factor of Activated T cells (NFAT) family of transcription factors^4,5^.

NFAT family includes four classic members: c1, c2, c3 and c4 which were first described in immune cells^4,5^ and have been associated with malignancies and tumour progression (reviewed in^6,7^). NFATc members expression has been shown in astrocytoma^8^, C6^9^ and U251 glioma cell lines^10^. Inhibition of the Ca/CN/NFATc pathway by cyclosporine A (CsA) and FK506 has a negative impact on the growth/survival rate of rat and human GBM cells and on glioma invasion capacity^11,12^. Accordingly, CsA infusion inhibits tumour growth of implanted glioma *in vivo*^11^. Although the relevance of the Ca/CN/NFATc pathway in glioma biology has been already described by using pharmacological intervention, the mechanism and contribution of the different individual NFATc members, and their transcriptional program, remain to be fully assessed.

Cytokines have been proven to be potent mediators of antitumor immunity, and overall, their use as treatment for glioma has produced mixed results. The initial preclinical successes using systemic or local delivery of recombinant cytokines failed to adequately translate into clinical benefit^13^, but the search for possible cytokine or other immune based therapies remains to be fully explored. Cytokines and cytokine receptors expressed by glioma cells have been shown to modulate cell invasiveness, survival, proliferation and angiogenesis^14^, and many of these cytokines are over-expressed in high grade human glioma^15^. Cytokines and chemokines prevalently produced in glioma tissue include Tumour Necrosis Factor alpha (TNF-α)^16^, IL-8 (CXCL8)^17–19^, macrophage chemo attractant protein-1 MCP-1/CCL2^20^, granulocyte-macrophage colony-stimulating factor (GM-CSF/CSF2)^21^ and Chemokine Receptor 3 (CXCR3). In other cellular systems, the expression of these cytokines has demonstrated to be sensitive to the inhibitors of the Ca/CN signalling pathway such as CsA^22–25^. The source of these molecules, attributable to glioma cell, surrounding glial cells, tumour-associated macrophages (TAMS), neuronal or lymphocytes, remains to be fully elucidated.

In this work, we evaluated the expression of the individual NFATc members and investigated the role of the most abundant, NFATc3, by exogenous activation and knock-down strategies. We showed that NFATc3 has a positive role in tumour growth, since NFATc3 knock-down inhibits both proliferation and migration of U251 glioma cells as a model. Our results suggest that NFATc3 alone is able to modulate a cytokine network required for the normal growth of glioma. Tools limiting NFATc3 might be considered useful to explore future therapeutic approaches against glioma.

## Results

### Analysis of NFATc member expression in U251

CN/NFATc pathway has been previously implicated in the regulation of glioma growth by using pharmacological inhibitors^9,26^. Nevertheless, the expression and the contribution of the different NFATc members in glioma cells have not been completely assessed.

**Figure 1:**
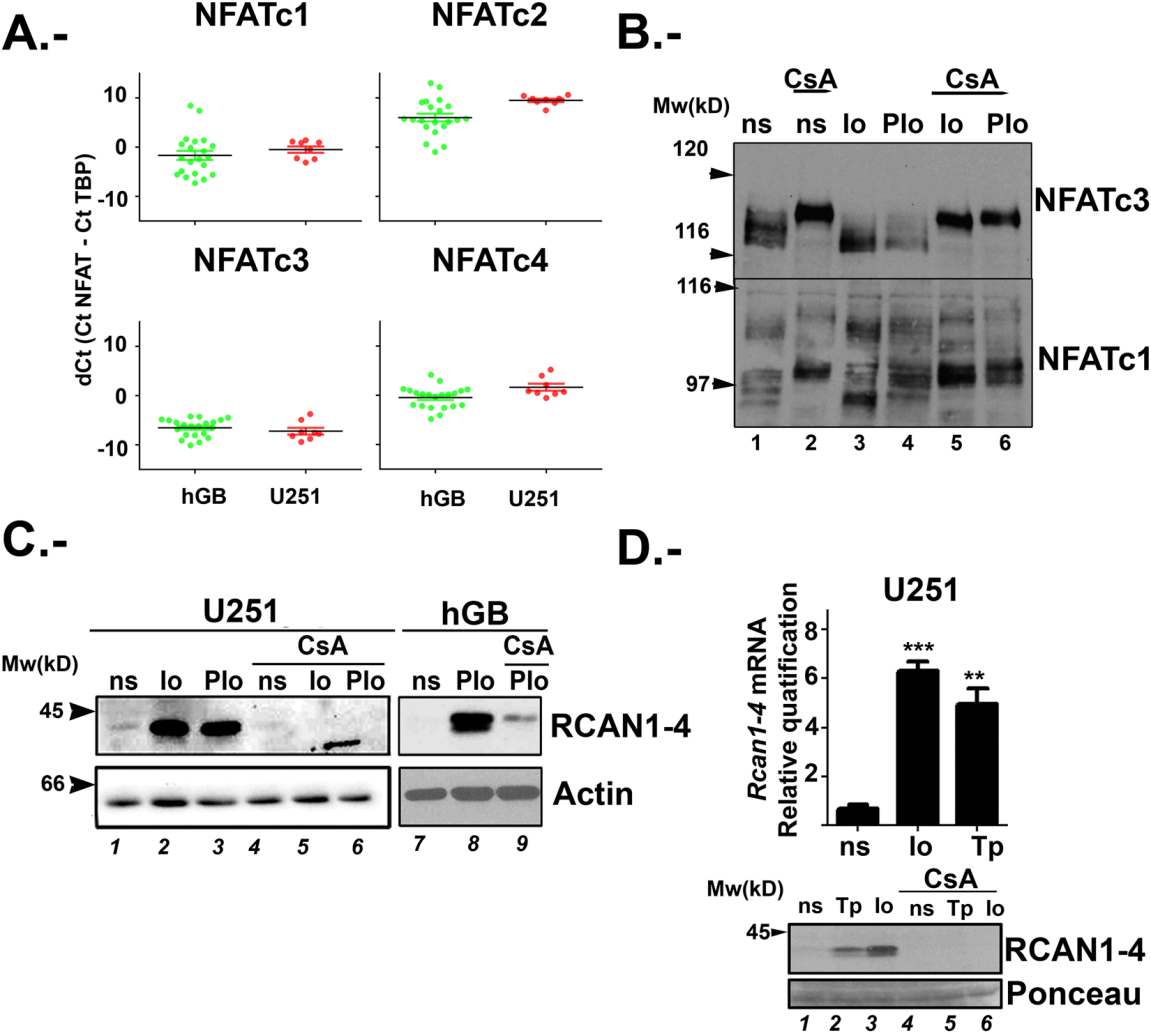
Analysis of NFATc expression and calcium/Calcineurin/NFAT signalling in glioma cells. (**A**) *NFATc1*, *NFATc2*, *NFATc3*, and *NFATc4* mRNAs from U251 and different human Glioblastoma lines (hGB) from xenografts were amplified by TaqMan RT-PCR. *NFATc* mRNA was normalized to the expression of TBP as endogenous gene. Results are shown as dCt (Ct NFATc − Ct TBP). (**B**) Representative immunoblot showing endogenous expression of NFATc3 and NFATc1 in U251 total protein lysates. Cells were 1 hour pre-treated with 200 ng/mL CsA (lanes 2, 5 and 6) and then, non-stimulated (ns) as control or stimulated for 30 minutes with 1 μM ionophore alone (Io) or in combination with 20 ng/mL PMA (PIo). (**C**) Representative immunoblot showing endogenous RCAN1-4 protein expression. β-actin expression was used as loading control. Glioma total protein lysates from U251 or hGB were pre-treated without (lanes 1 to 3) or with CsA (200 ng/mL) (lanes 4 to 6) and then stimulated for 4 hours with Io (1 μM) or in combination with PMA (20 ng/mLPIo as indicated. (**D**) In the upper panel, RCAN1-4 mRNA was amplified from total RNA by TaqMan RT-PCR. U251 were exposed 4 h to vehicle, Io (1 μM) or thapsigargin A (Tp, 10 nM). RCAN1-4 mRNA was quantified in arbitrary units normalized to the expression of human TBP. Representative experiments of a minimum of three are shown; values are the mean ± SD of triplicate RT-PCR determinations for each condition. ***P < 0.001; **P < 0.01 (ANOVA) versus ns that was given a value of 1. Panel below, representative immunoblots for RCAN 1-4 protein expression with ponceau staining as loading control in U251 cells treated as above (n = 3).

U251 is a cell line widely established as a model for human glioma of astrocytic nature^27^. First, we evaluated the expression of the individual NFATc members (c1 to c4) by quantitative PCR (qPCR) in U251 cells, together with a collection of human glioblastoma (hGB) cell lines obtained from xenografts. We found that *NFATc3* has the highest expression levels in both hGB cell lines and U251. *NFATc1* is also clearly expressed, but in relative less amount. *NFATc2* transcript was observed only in some hGB cell lines but hardly detected in U251, and *NFATc4* expression varied among hGB cell lines (Fig. 1A), while it was detectable only in starved U251 (data not shown). In summary, U251 cells have a NFATc expression pattern similar to most of the primary hGB tested, and *NFATc3* and *NFATc1* are consistently expressed in both models.

Figure 1A shows mRNA expression data presented as delta Ct (dCt). dCt corresponds to the difference between the Ct (cycle threshold) of target gene and the Ct of the reference gene, i.e. TBP (TATA binding protein). By using TaqMan probes with similar efficiency, NFATc3 dCt negative values imply that this member is the most abundant in glioma samples. Our results are consistent with data generated by the TCGA Research Network showing that *NFATc1* and *NFATc3* are expressed in higher amount in human glioblastoma samples when compared to normal brain tissue. On the other hand, *NFATc2* and *NFATc4* expression was comparable between normal and tumour samples (Supplemental Fig. S1A), although with low abundance (Fig. 1A). Furthermore, we interrogated the RNAseq data of already published results (TCGA-GBM study), and found that there was a significant increase of *NFATc* expression across glioblastoma tumour grade (Supplemental Fig. S1B).

Then, we evaluated individual NFATc1-c4 members expression at the protein level using specific antibodies previously validated^28,29^. U251 total protein extracts showed clear NFATc3 and NFATc1 protein expression (Fig. 1B); NFATc2 and NFATc4 immunoblots did not reveal specific signal of the expected molecular weight. NFATc proteins have a complex electrophoretic mobility since there is different phosphorylation/ dephosphorylation status of NFATc proteins in response to changes in intracellular calcium concentration ([Ca^2+^]i) by ionophore A23187 (Io)^30^. Cells were stimulated with Io (1 μM) for 30 minutes and, as expected, faster migrating bands were detected, corresponding to dephosphorylated forms of NFATc3 and NFATc1 (Fig. 1B, lane 3). Noteworthy, NFATc1 antibody recognized different NFATc1 isoforms, as previously described^29^. As additional control, CsA pre-treatment, known to inhibit the CN dependent NFATc dephosphorylation, retarded the gel mobility of NFATc3 and NFATc1 members, confirming antibody specificity (Fig. 1B, lanes 5 and 6).

Therefore, we consider that U251 is a valuable glioma model since the expression pattern of NFATc members is comparable to other hGB cell lines and clinical samples (Fig. 1A and Supplemental Fig. S1) and confirmed the specificity of the antibodies used.

### RCAN1-4 gene is induced in U251 glioma cells in a Ca/CN/NFATc dependent manner

RCAN1-4 is a member of the calcipresin family, endogenous modulators of CN activity (reviewed in^31^), known to be involved in tumour progression^32^. We and others have previously shown that RCAN1-4 expression is highly sensitive to NFATc activation. Therefore, it has been used as a sensor for the Ca/CN/NFAT pathway activation in different biological setting^30,33,34^. Thus, we tested if an increase of [Ca^2+^]i by Io alone was able to induce the RCAN1-4 expression in U251 glioma cells. We also included, as stimulus, the MAPK inducer Phorbol 12-myristate-acetate (PMA) in combination with Io (PIo) to activate other cofactors known to partner with the NFATc proteins to regulate a variety of genes^29,30^. We observed that Io alone or in combination with PMA (PIo), increased RCAN1-4 protein levels. This increase was inhibited by CsA pre-treatment (Fig. 1C). Alternative glioma cell lines, such as primary hGB and U87 showed similar results (Fig. 1C and data not shown). Accordingly, analysis by qPCR showed that Io treatment induced the accumulation of *RCAN1-4* mRNA in a CsA sensitive manner (Fig. 1D). Similar results were confirmed by using other means of increasing [Ca^2+^]i such as thapsigargin A (Tp) (Fig. 1D). When U251 glioma cells were stimulated with PMA alone, there was no detectable induction of RCAN1-4 mRNA or protein, in accordance to the results previously observed in primary astrocytes^30^ (Supplemental Fig. S2).

### NFATc3 protein is the main modulator of RCAN1-4 expression in glioma

**Figure 2:**
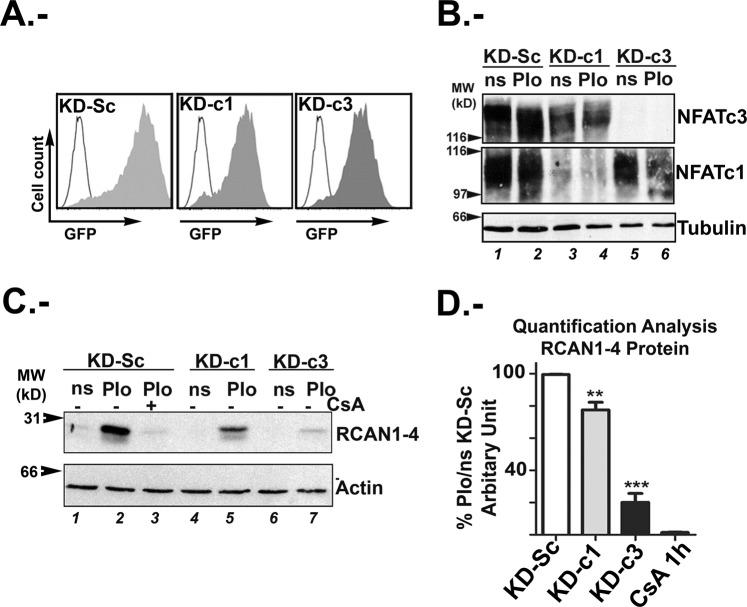
Specific knockdown of NFATc proteins modulates RCAN1-4 expression. (**A**) Representative infection efficiency monitored by GFP expression using flow cytometry. U251 cells were transduced with control scramble (KD-Sc), NFATc1 (KD-c1) and NFATc3 (KD-c3) shRNA lentiviral vectors (LV) as in material and methods. (**B**) Representative immunoblots showing endogenous NFATc3 and NFATc1 proteins from shRNA-transduced U251 cells. Cells were stimulated with PMA (20 ng/mL) plus Io (1 μM) for 1 hour. Expression of α-tubulin as loading control was used. (**C**) Representative Immunoblots showing endogenous RCAN 1-4 protein expression, from KD-Sc, KD-c1 and KD-c3 shRNA U251cells treated for 4 hours with Io (1 μM) in combination with PMA (20 ng/mL), PIo. β-actin expression was used as loading control. (**D**). Quantification analyses of 4 h PIo induced RCAN 1-4 protein expression in KD-Sc, KD-c1 and KD-c3 U251 cells. One hour CsA (200 ng/mL) pre-treatment of PIo stimulated U251 was used as control for efficient inhibition of RCAN1-4 protein expression. Band Intensity of immunoblots were analysed by densitometry, normalized to β-actin levels and represented as the mean ± SD of the fold change from non-stimulated (ns) KD-Sc cells that was given the value of 100%; (n = 4). ***P < 0.001; **P < 0.01 (ANOVA).

Since NFATc members have been associated with malignancies and tumour progression (reviewed in^6^), we investigated the individual contribution of NFATc members expressed in U251 glioma cell line. Therefore, we used lentiviral vectors expressing short hairpin RNA (shRNA) to specifically silence each of the two NFATc members mostly expressed in these cells. Lentiviral shRNA vectors targeting NFATc1 and NFATc3, defined as NFATc1 shRNA (KD-c1) and NFATc3 shRNA (KD-c3), and lentiviral vectors encoding for a scramble shRNA (KD-Sc) as control, were previously described and validated^29^. More than 95% infection efficiency was monitored by flow cytometry of Green Fluorescent Protein (GFP) reporter gene (Fig. 2A). Immunoblot analyses showed that KD-c3 yielded a very efficient silencing of NFATc3 protein, although a minimal effect on NFATc1 expression was also observed. When KD-c1 was used, NFATc1 knockdown was also efficiently achieved with minimal inhibition of the NFATc3 expression. This slight cross-downregulation between specific shRNA and non- targeted NFATc member might be due to either mechanisms of transcriptional cross regulation among members or, giving the high efficiency of infections, to shRNA unspecificity (Fig. 2B). This effect have not been observed in other cell types^29^. Besides the minimal cross inhibition between shRNA and NFATc members, considering the strong knockdown effect on the target member, we assumed that most of the experimental effects observed were consequence of the target member silencing. Next, we analysed the effect of NFATc1 and NFATc3 knock-down on RCAN1-4 protein expression. PIo-induced RCAN1-4 protein expression was inhibited by 80% in KD-c3 cells when compared to KD-Sc control and the effect was comparable to the CsA pre-treatment (Fig. 2C,D). A lesser, but significant, inhibition was also observed in KD-c1 cells (Fig. 2C,D). In this case, seen the small reduction, we did not discard the possibility that this effect might be associated to the slight NFATc3 inhibition by KD-c1 and not strictly to NFATc1, but further analyses will be needed.

In summary, our data suggest that NFATc3 is highly expressed in human glioma and silencing of this member is critical for RCAN1-4 expression in U251.

### Analysis of genes regulated by NFATc3 in U251 glioma cells

**Figure 3:**
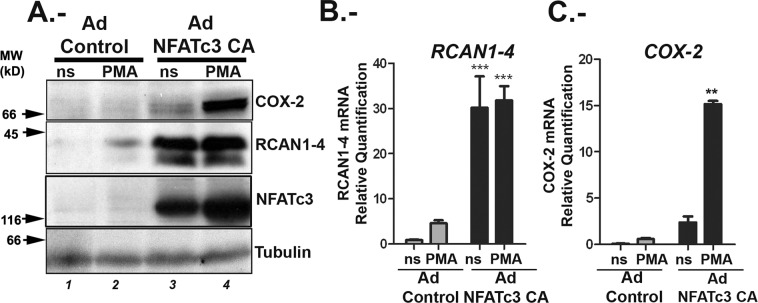
Expression of constitutively active NFATc3 supports COX-2 and RCAN 1-4 mRNA and protein expression in glioma cells. U251 cells were transduced with either adenovirus control (Ad Control) or encoding a constitutively nuclear NFATc3 (Ad NFATc3 CA). After 48 hours cells were quiesced and then stimulated (4 h) with PMA (20 ng/ml) (**A**) Representative immunoblots of 3 independent experiments showing protein expression of COX-2 (upper panel), RCAN1-4 (middle panel) and NFATc3 (lower panel). α-Tubulin was detected as loading control; molecular weights (MW kD) are indicated to the left. (**B**,**C**) Quantitative RT PCR of RCAN1-4 and COX-2 mRNA expression from Ad Control and Ad-NFATc3 CA, non- stimulated (ns) and PMA-stimulated U251 cells. Levels are presented as the fold expression above ns cells. Values are means ± SD of RT-PCR determinations for each condition (n = 4). Identical concentrations of adenoviral particles were used for each infection in all experiments. TFRC and GAPDH were used as endogenous control, ***P < 0.001; **P < 0.01 (ANOVA) versus ns.

To define cancer related genes depending on NFATc3 activity in glioma cells, we infected U251 with an adenovirus encoding nuclear constitutively active (CA) NFATc3 (Ad NFATc3 CA)^35^, or with an empty adenovirus vector (Ad Control) as a mock control. We have shown in astrocytes that RCAN1-4 depends on NFATc activity alone, while COX-2 (Cyclooxygenase 2) transcription depends on a cooperation between NFATc and cofactors activated by stimulation with PMA^30^. COX-2 and RCAN1-4 protein expression was used as read out to validate the constitutive activation of this signalling pathway with the Ad NFATc3 CA (Fig. 3A). Cells were left non stimulated (ns) or stimulated with PMA alone. Since the Ad NFATc3 CA is a constitutively nuclear localized mutant, calcium signal is not required to activate NFATc3. As expected, the presence of only nuclear NFATc3 was sufficient for maximal RCAN1-4 protein and mRNA expression (Fig. 3A,B); whereas COX-2 expression was maximally induced after stimulation with PMA of U251 infected with Ad NFATc3 CA (Fig. 3A,C), coherent to published data on astrocytes^30^. We have noticed that in some of the experiments the adenoviral vectors alone increased slightly the amount of RCAN1-4 protein and mRNA, mainly in PMA treated cells (Fig. 3A,B). Adenoviral gene transfer could result in alteration in membrane structure and in turn can affect intracellular [Ca^2+^]i^36^. In fact, PMA-stimulated induction of RCAN1-4 was not statistically different between experiments and, most importantly, this increase was not observed in cells treated with PMA but not transduced with adenoviral vectors, as shown in supplemental Fig. 2S.

**Figure 4:**
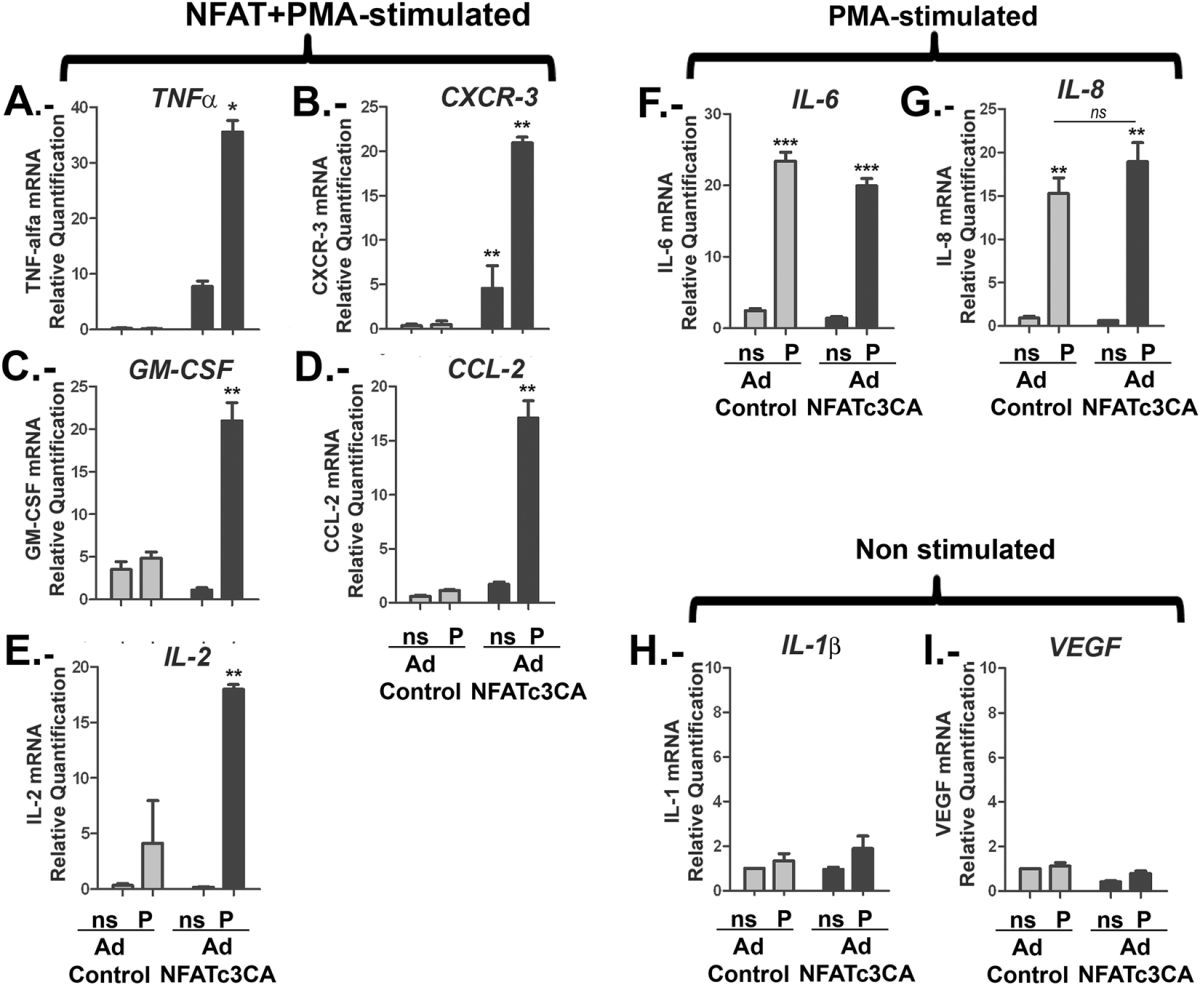
NFATc3 nuclear expression up-regulates cytokines with a prevalent role in glioma biology. U251 were infected with adenovirus encoding constitutively-active NFATc3 (Ad NFATc3) or control adenovirus (Ad Control). After 48 hours cells were quiesced and then stimulated (4 h) with PMA (20 ng/ml). For each adenovirus, nine independent experiments were performed and mixed in three pools to be analysed. cDNAs were obtained from each pool and specific mRNA expression for each cytokine was assayed by RT-PCR using the probes described in material and methods. Levels are presented as the fold expression above non-stimulated cells (ns) and TFRC and GAPDH were used as endogenous control **P < 0.01, *P < 0.05 with respect to non-stimulated (ns) Ad control-infected U251 cells values were given a value of 1. (**A**–**E**) Quantitative RT-PCR of NFATc3 CA above PMA regulated genes: TNFα; CXCR-3; G;-CSF; CCL2 and IL-2. (**F**,**G**) Quantitative RT-PCR of PMA but not NFATc3 CA- regulated genes (IL-6 and IL-8). (H and I) Quantitative RT-PCR of non PMA- or NFATc3 CA-regulated genes (IL1β and VEGF).

To understand if NFATc3 is a critical factor for cytokines and chemokines known to be involved in tumour growth, we evaluated the impact of nuclear NFATc3 on *TNF-α*, *CXCR-3*, *GM-CSF*, *IL-2 and CCL-2 mRNA* expression. As shown in Fig. 4A–E, the Ad NFATc3 CA expression alone increased *TNF-α*, *CXCR-3*, *GM-CSF*, *IL-2 and CCL-2* mRNA levels. PMA stimulation was needed to achieve maximal induction of these genes, indicating the requirement of cofactors that are elicited by other signalling pathways, similarly to *COX-2* (Fig. 3). IL-6 and IL-8 were included as controls, representing genes expected to be mainly regulated by PMA-dependent signals. In fact, IL-6 promoter is known to contain binding sites for several transcription factors which contribute to the complex regulation of this gene^37^. For IL8 promoter, participation of a number of different transcription factors has also been described: nuclear factor-κB (NF-κB), activating protein (AP-1), CAAT/enhancer-binding protein β (C/EBPβ, also known as NF-IL-6), C/EBP homologous protein (CHOP) and cAMP response element binding protein (CREB)^38^. In our experiments, the presence of PMA alone was sufficient to obtain maximal expression of *IL-6* (Fig. 4F) and *IL-8* (Fig. 4G) mRNA. However, a relevant role for NFATc in IL-8 expression has been described in some other type of cancerous cells^39^. In our results, the fold induction of *IL-8* mRNA in presence of PMA was of 17.15 ± 1.89 over the control cells, while in presence of NFATc3 CA the fold induction was only mildly increased to 20.51 ± 1.13, a difference that was not statistically significant. *IL-1β* and *VEGF* were included as negative control for all the stimuli tested, as shown in Fig. 4H,I.

**Figure 5:**
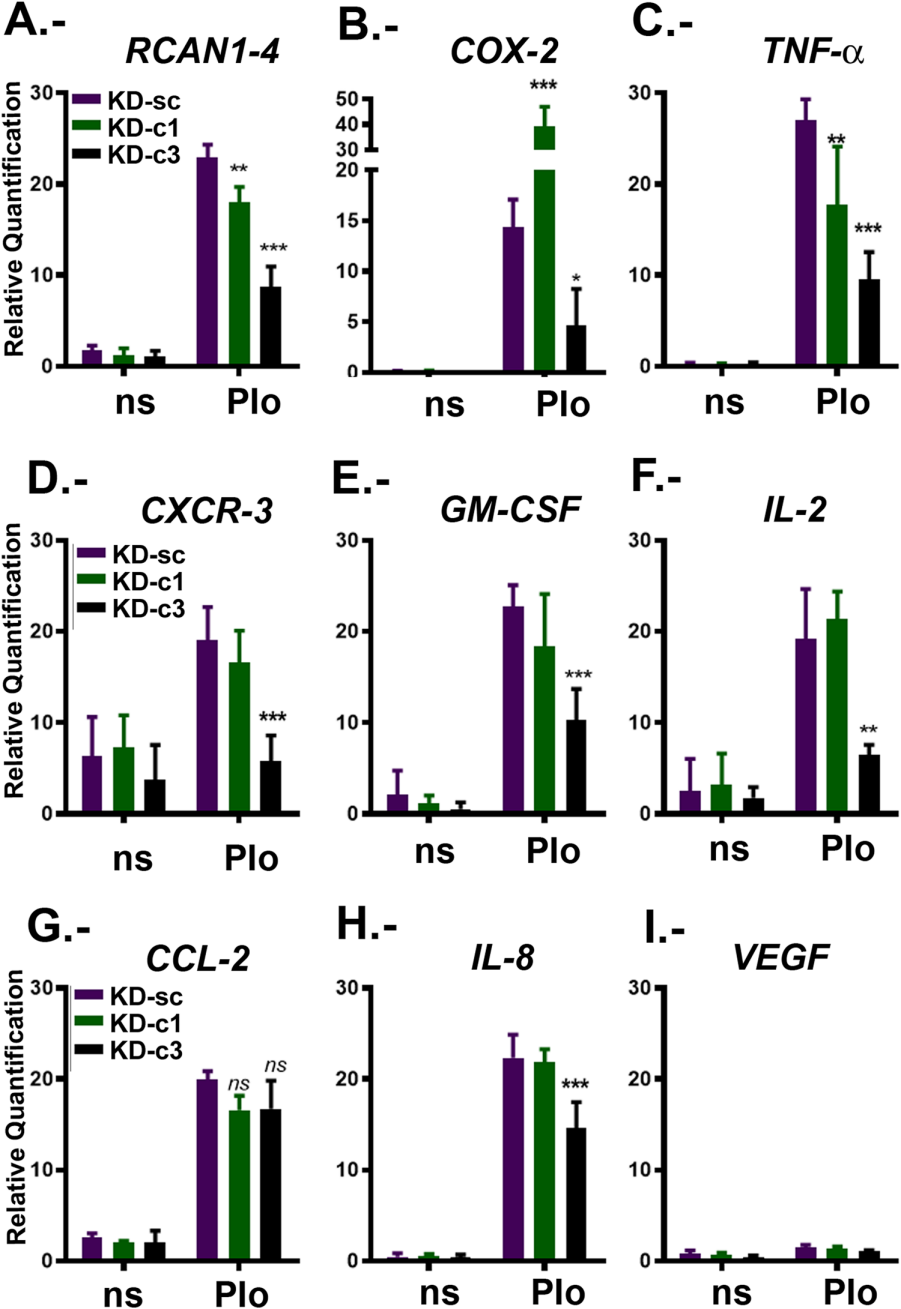
Gene expression regulation by NFATc3 and NFATc1 silencing in glioma cells. KD-Sc, KD-c1 and KD-c3 U251 cells were non stimulated (ns) or 4 h treated with 1 μM Io in combination with 20 ng/mL PMA (PIo). (A to I) RCAN1-4; COX-2; TNFα; CXCR3; GM-CSF; IL-2; CCL2; IL-8 and VEGF mRNAs were quantified and normalized to the expression of TFRC and GAPDH as endogenous control. Levels are presented as the fold expression above non-stimulated cells (ns that was given a value of 1 in arbitrary units). Values are the mean ± SD of triplicate RT-PCR determinations for each condition. ***P < 0.001; **P < 0.01; *P < 0.05 (ANOVA) with respect to PIo stimulated KD-Sc cells.

To further explore the impact of NFATc3 in the regulation of these genes, we compared control KD-Sc cells with silenced KD-c3 cells. PIo-treated KD-Sc cells showed an increase in the levels of *TNF-α*, *CXCR-3*, *GM-CSF*, *IL-2*, *CCL-2; IL-8* (Fig. 5C–H) similar to PIo-treated non transduced cells (data not shown). NFATc3 silencing significantly impaired the expression of PIo dependent genes *RCAN1-4*, *COX-2; TNF-α; CXCR-3*, *GM-CSF and IL-2* (Fig. 5A–F). Unexpectedly, PIo-induced *CCL-2* expression was not affected (Fig. 5G) and we observed a mild but consistent inhibition of *IL-8* mRNA induction in KD-c3 cells (Fig. 5H), that might suggest some NFATc role as previously described in other tumour cells^39^. *VEGF* was not induced by PIo treatment nor inhibited in KD-c3 cells (Fig. 5I).

NFATc members are known to bind the same sequence on target genes promoters. Although we found that NFATc3 is the major member expressed in glioma cells we wanted to evaluate if NFATc1 has a role for the expression of these cytokines (Fig. 5). We found that NFATc1 silencing had no impact on *CXCR-3*, *GM-CSF*, *IL-2 and IL-8* mRNA expression and concluded that these genes are NFATc3 dependent in this cellular setting. Nevertheless, our data indicated that NFATc1 could participate in the *RCAN1-4* (as shown in Fig. 2C,D) and *TNF-α* regulation (Fig. 5). Finally, NFATc1 seems to be relevant for the regulation of COX-2 but as negative regulator, in contrast to NFATc3. NFATc1 as a negative regulator has been previously described in lymphoid cells^29^ and muscle cells^40^.

### NFATc3 controls U251 tumour growth in an orthotopic mouse model

**Figure 6:**
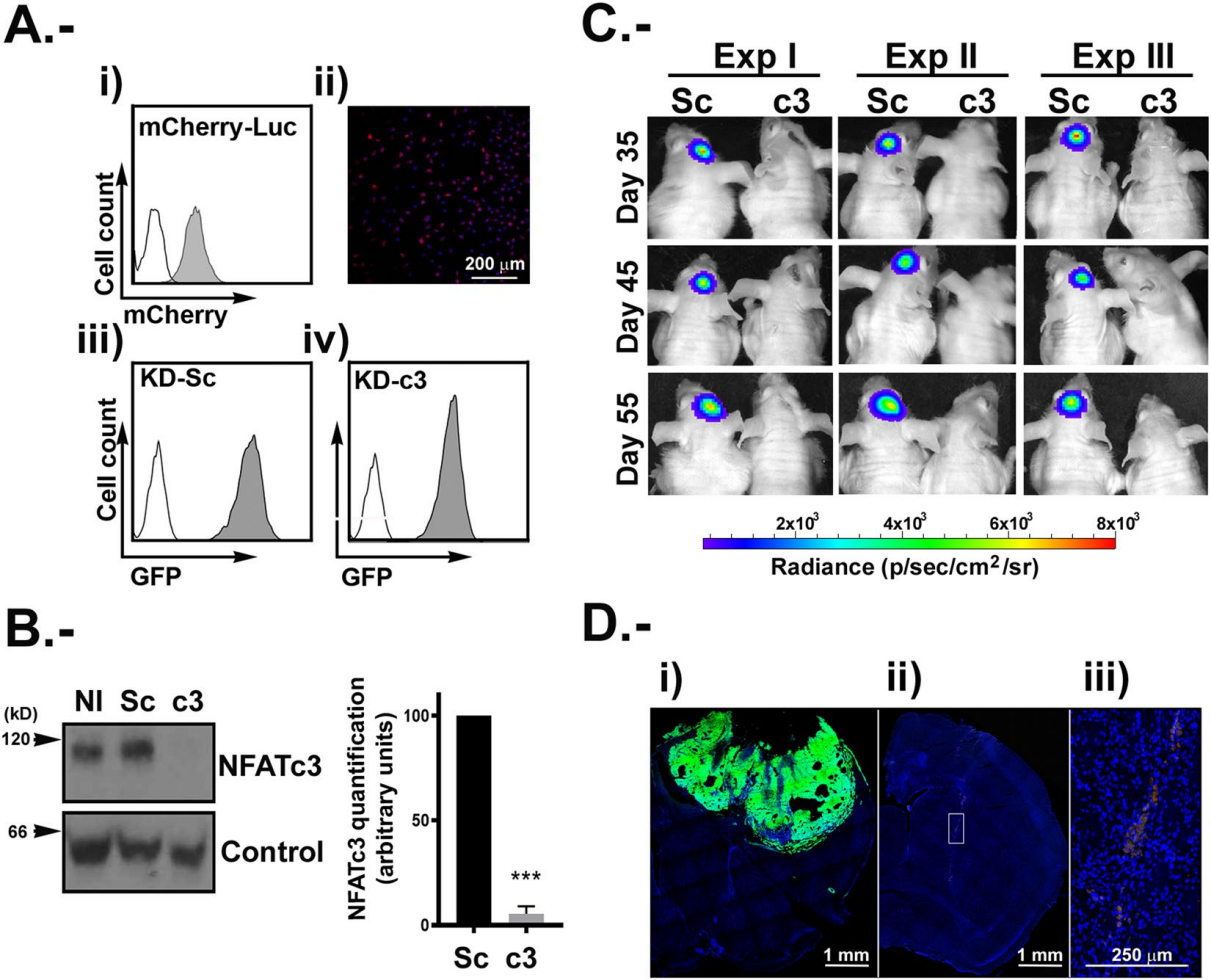
Tumour growth inhibition *in vivo* by NFATc3 silencing. U251 mCherry-Luc expressing mCherry protein and Luciferase markers was used (**A**) Panel i, analysis of cherry positive cells by flow cytometry (grey histograms) using non-transduced U251 cells (white histogram) as control. Panel ii, representative microphotograph of confocal microscopy image of U251mCherry cells. iii) and iv) U251 mCherry-Luc were transduced with KD-Sc and KD-c3 shRNA lentiviral vectors expressing GFP. Flow cytometry analyses of GFP positive cells in grey histogram. (**B**) Representative immunoblotting of endogenous NFATc3 from U251 mCherry-Luc cells KD-Sc and KD-c3 used in the orthotopic mice model, together with the densitometry analyses showing efficient blockage of NFATc3 expression. Quantification analysis of NFATc3 in U251 mCherry-Luc cells KD-Sc and KD-c3 used in the orthotopic mice model. Band Intensity of immunoblots were analysed by densitometry, normalized to control bands and represented as the mean ± SD of the fold change from non-stimulated KD-Sc cells that was given the value of 100%; ***P < 0.0001 (t-test). Data obtained from 4 different experiments. (**C**) Bioluminescence intensity for “*in vivo*” luciferase activity (n = 3), from mice injected with U251 mCherry-Luc–KD-Sc (Sc) and U251 mCherry-Luc-KD-c3 (c3) days 35, 45 and 55. Mice injected with c3 cells did not show detectable bioluminescence by IVIS analyses. (**D**) Representative microphotograph showing direct GFP fluorescence map of brain coronal sections injected with Sc (panel i) and c3 (panel ii) cells after 55 days. Panel iii shows digital magnification of GFP positive cells in c3 injected mice. Images were obtained with a SP5 Leica TCS confocal fluorescent microscope. Scale bars are included.

Our results indicate that NFATc3 is the most expressed NFATc member in U251 and other hGB cell lines and in an array of human glioma samples according to published databases (Fig. 1 and Supplemental Fig. 1S). In addition, we suggest here that NFATc3 is a key positive regulator of *RCAN1-4*, *COX-2; TNF-α; CXCR-3*, *GM-CSF* and *IL-2*, known to be implicated in glioma growth^15–21^. Therefore, we evaluated the specific contribution of this NFATc member in tumour formation *in vivo*. We established a reporter U251 cell line expressing the fluorescence marker mCherry and the luciferase gene, selected based on puromycin resistance (U251 mCherry-Luc). Next, puromycin-selected mCherry-Luc U251 glioma cells were transduced with KD-Sc (Sc) or KD-NFATc3 (c3) lentiviral vector. Cells were analysed by flow cytometry confirming that 100% of the cells co-expressed mCherry and GFP proteins (Fig. 6A). Before injection, NFATc3 knockdown was validated by immunoblotting (Fig. 6B).

In order to evaluate if the knockdown of NFATc3 has an impact on the viability of the glioma, we performed an orthotopic glioma mouse model, previously described in^41^. Briefly, 1 × 10^5^ of U251 Sc or c3 were injected in the striatum of nude mice. Luciferase expression was assessed as a proxy of tumour growth every 10 days, starting from day 35, until the end of the experiment (55 days), when control Sc animals started to show physical distress. Luminescence signal showed that implanted Sc tumour cells were surviving and growing. In contrast, implanted c3 tumour cells was under the detection limit. As a further confirmation that c3 cells were not able to grow *in vivo*, we visualized by immunofluorescence GFP expression in brains isolated from the two animal groups (Fig. 6D). The results confirmed the luciferase measures: animals injected with c3 cells did not show glioma growth. Interestingly, the surviving c3 cells were observed sitting around the injection area, although they did not appear to be able to grow or invade the surrounding tissue (insert in Fig. 6D).

### NFATc3 is required for U251 proliferation and migration

There are two main cellular pathways involved in the establishment of tumour mass: proliferation and migration. Migration and invasion of glioma cells is a very complex and multistep process that comprises the coordinated action of different cytokines and enzymes^42^. As mentioned above, we have found that NFATc3 is required for the expression of several mediators known to be involved in tumour growth (Figs 3–5). Additionally, it has been previously demonstrated that the inhibition of the whole NFATc signalling pathway by CsA is able to inhibit the invasion capacity and growth of glioma cells^12^. Therefore, we questioned the specific contribution of NFATc3 to this process by analysing the effect of NFATc3 silencing in the U251 glioma growth *in vitro*.

**Figure 7:**
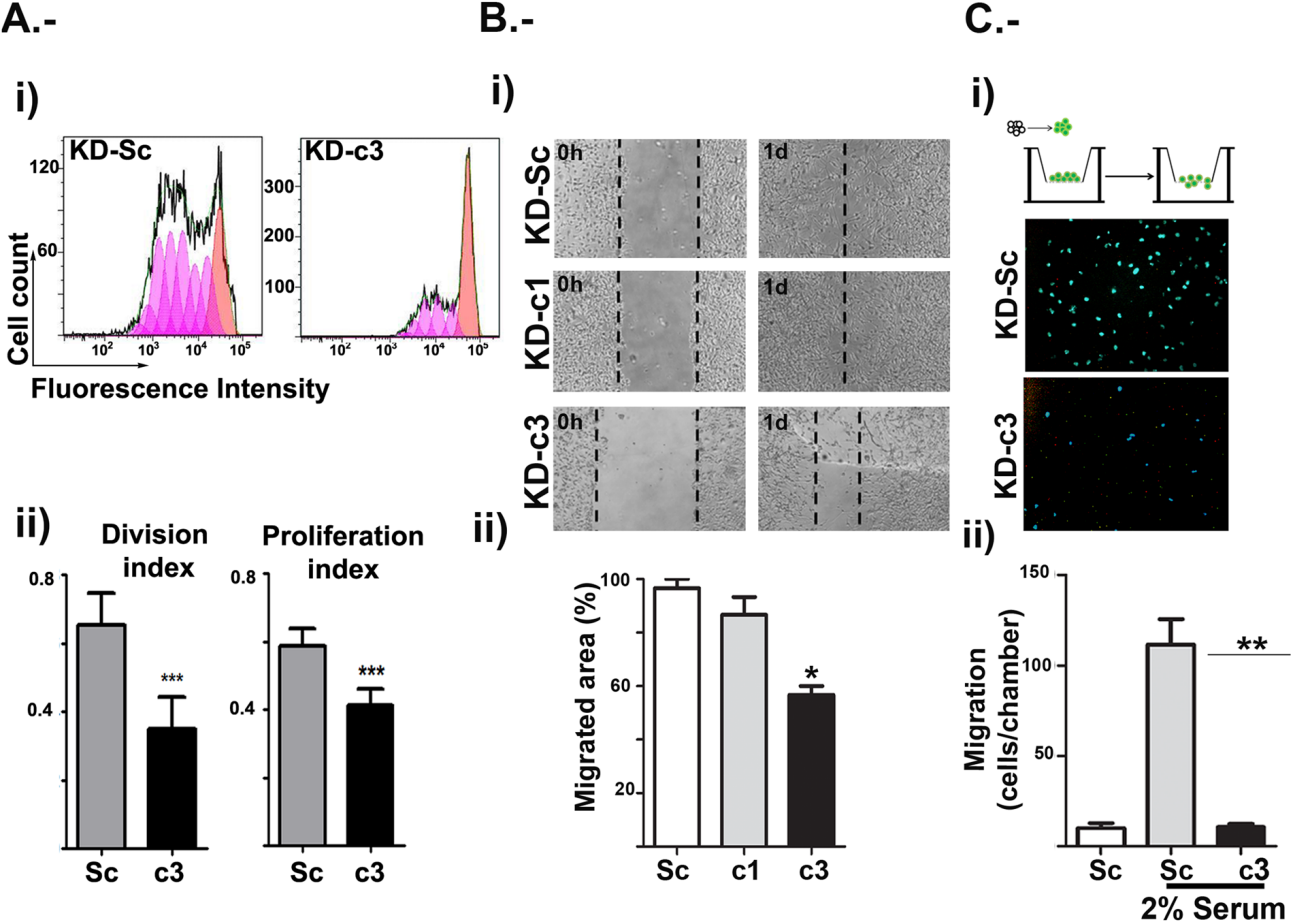
NFATc3 is required for glioma proliferation and migration. U251 cells were transduced with control (KD-Sc), NFATc1 (KD-c1) and NFATc3 (KD-c3) shRNA lentiviral vectors (LV) as before. (**A**) KD-Sc and KD-c3 cells were labelled with Violet proliferation Dye 450 and left in 2% FBS media. Cells were analysed by flow cytometry 1 and 3 days after treatment. Panel I cytometry histograms showing dilution of fluorescent compound in each division. Panel ii Division index: as the average number of cell divisions that a cell in the original population has undergone, and Proliferation index: the total number of divisions divided by the number of cells that went into at least one division. A representative experiment is shown in panel i, and indexes are measures of 4 independent experiments. ***P < 0.001 with respect to index in KD-Sc cells. (**B**) Wound-healing assay from KD-Sc, KD-c1 and KD-c3 U251. Panel i, photomicrograph of representative fields at initial time (0 h) and after 24 hours (1d) 2% FBS stimulation. Dotted lines mark the limits of the unpopulated area. Panel ii shows quantification of migrated area (the proportion of the denuded area repopulated by migrating cells) at 24 hours (mean ± SD; n = 3). *P < 0.05 with respect to KD-Sc cells. (**C**) Transmigration assay was carried out using Boyden chamber assays (scheme on panel i). KD-Sc and KD-c3 cells were platted on the Transwell inserts and 2% FBS was added to the lower chamber. After 4 hours, non-migrated cells were washed and fixed as in material and methods. Panel i show microphotographs of randomly chosen fields of paraformaldehyde fixed cells stained with Hoechst 33342 to visualize nuclei. Panel ii shows quantification of migrating cells per chamber. Data are shown as the mean ± SD of three independent experiments. **P < 0.01 with respect to KD-Sc cells without FBS in the lower chamber.

The proliferation capacity of KD-Sc and KD-c3 cells was measured by flow cytometry during three days. As shown in Fig. 7A, the NFATc3 knockdown markedly reduced the proliferation capacity of U251 cells in presence of low serum (2%), although KD-Sc cells, in the same conditions, were able to proliferate (Fig. 7A).

Next, considering that the cells inoculated in the mouse model appeared to have remained in the same site of injection, we decided to investigate the effect of NFATc3 knockdown on the migration capacity of U251 glioma cells *in vitro*. U251 were grown to confluence and a wound-healing assay was performed by linear disruption of the monolayer. As additional control, KD-c1 cells were also included. 24 h after the monolayer disruption, KD-Sc and KD-c1 U251 cells efficiently reoccupied the denuded area, while KD-c3 cells were unable to produce the closure, indicating impaired proliferation and migration capacity (Fig. 7B).

The migratory activity of U251 cells was further assessed by transwell assay in response to a serum gradient. After 4 hours, the number of KD-c3 cells migrating through the membrane of the transwell chamber was reduced when compared with KD-Sc cells, indicating the strong participation of NFATc3 in the glioma cells migration (Fig. 7C).

Therefore, our results show that NFATc3 expression is required for both proliferation and migration and are in line with the data obtained in the *in vivo* model.

## Discussion

Glioma therapy without damaging the affected brain parenchyma is still difficult due to the infiltrative growth pattern of these tumours and the rapidly growing rate of this type of cancer cells. The Ca/CN/NFATc pathway has been used as a target for various inhibitors, with discrete success and large side effects. If it would be possible to block this route more specifically, better therapies could be developed without the inconveniences presented by the current ones.

Our data suggest that *NFATc1* and especially *NFATc3* are overexpressed in tumour samples compared with normal tissue, while *NFATc2* is minimally expressed. This result is not in line with the study from Tie *et al*.^43^ where *NFATc2* is described as highly expressed. The reason behind this apparent contradiction might be related to the selection of high grade glioma samples in the referred study. TCGA data show that all members are overexpressed in grade IV glioma samples, and our data suggest a role of *NFATc3* in earlier phases, during the establishment of the tumour.

The U251 glioma model used in this study meets a number of requirements: these cells are of astrocytic nature, which account for 60–70% of total malignant brain tumours and has been used widely to study the effect of different signalling pathways involved in the orthotopic intracranial assays. Here, in the context of *NFATc* expression, we have validated this model by showing that U251 reproduced a pattern of *NFATc* expression similar to primary hGBs cell lines and glioma samples (TCGA-GBM data). Nonetheless, we found that the expression of the individual *NFATc* members varies in the different human xenografts lines tested, being always *NFATc3* the most abundant (Fig. 1). This indicates that to interfere efficiently with the NFATc-signalling, an analysis of the different members in the distinct type of glioma cell should be carefully performed.

Analysis of NFATc proteins is a difficult task, as mentioned above. Here we describe the appearance of slower migrating bands for NFATc3 in response to 1 h PIo treatment in line with previously reported by our group in other cells^29,30,44^. Other NFATc members, such as NFATc2, have a similar behaviour. Pre-treatment of these cells with CsA, retards the gel mobility of all NFAT members analysed, as previously described^28,29^, and is shown here in Fig. 1B.

RCAN1-4 has been previously shown to be a NFATc-target gene in a variety of cellular systems^30,34,45^, sensitive to calcium stress in human glioma cell lines^46^ and described as a sensor of the Ca/CN/NFAT signalling pathway activation in different biological setting^30,33,34^. In this study we have used pharmacological activation with Io or Tp alone to achieve a robust increase of [Ca^2+^]i. The physiological stimuli that produce [Ca^2+^]i increments has not been interrogated in this work, although there is a great variety of stimuli and receptors that have been involved in [Ca^2+^]i in glioma cells (reviewed in^47^ and references therein). The impact of CsA and FK506 as inhibitors of the Ca/CN/NFAT signalling pathway on glioma growth has been published before^9,26^. High doses of CsA (40 μM) has been reported to induces apoptosis of rat C6 glioma cells^48^. However, other authors have not found morphological changes indicative of apoptosis in human glioma cells line such as U251 when using a lower dose, 1 μM^49^. We have used 1 μM CsA which do not produce significant cell death but inhibit efficiently the activation of Ca/CN/NFAT pathway. Furthermore, KD-c3 and KD-Sc U251 cells present similar cell cycle profiles at 24 hours before injection in the orthotopic mouse model (Fig. 6 and data not shown).

Gene deletion of single NFATc members on immune cells has shown a mild impact, and this fact has been interpreted as the existence of functional redundancy (reviewed in^50^). Our experiments show that even in condition in which NFATc1 and NFATc3 knockdown were efficiently achieved, the majority of the NFATc-directed signalling correlates with NFATc3 presence. Combining the results obtained by silencing or over-expressing NFATc3, this study indicates that this member is involved in *RCAN1-4*, *COX-2*, *TNF-α*, *IL-2*, *GM-CSF* and *CXCR-3* gene regulation in U251 glioma cells. Although the KD-c3 reduced partially the expression of PIo-inducible *IL-8*, NFATc3 overexpression did not enhance *IL-8* induction above PMA values. It has been shown that IL-8 promoter can be activated by PMA-elicited signals alone in cancer cells^38^ although, in breast cancer, requires NFATc participation^39^. Therefore, it is quite plausible that the inhibition of the abundant NFATc3 in U251 might be able to partially compromise the IL-8 transcription in glioma cells.

Migration and invasion capacity of glioma cells are main features which account for the aggressive behaviour of brain tumours. Many processed are involved in tumour migration, from cytokine and chemokine expression, regulation of adhesion capacity and changes in the extracellular matrix. A role of NFATc signalling in migration has been described for different cancer cells^7^ (reviewed in^6^ and references therein) and, in other cell systems, has been related to the response to VEGF^34^, to SFRP (secreted frizzled-related protein)^51^ and activated Peroxisome Proliferator Activated Receptor gamma (PPARγ)^52^. Treatment of glioma cells with CsA inhibited growth of EGFP-transfected glioblastoma cells in organotypic brain slices^12^. CsA is an inhibitor of the CN, which has different cellular substrates besides NFATc^53^; if the CsA mediated inhibition of glioma proliferation and migration is NFATc dependent has not been clearly assessed *in vivo*. Here we show that NFATc3 is required for the normal proliferation and migration capacity of the U251 glioma cells, and for tumour establishment in xenografts.

We have identified NFATc3-dependent genes that are known to be involved in tumour migration: *COX-2* and *CXCR-3*. NFATc dependent *COX-2* induction has been observed in different cellular systems such as endothelial cells and breast epithelial cells and is required to promote invasive migration^29,54^. High COX-2 expression in tumour cells is associated with clinically more aggressive gliomas and is a strong predictor of poor survival^55^. More recently, epidemiologic studies have highlighted associations between the regular use of non-steroidal anti-inflammatory drugs (NSAID) and reduced glioma risks in humans and it has been shown that COX-2 pathway promotes gliomagenesis by directly supporting systemic development of myeloid derived suppressor cells (MDSCs) and their accumulation in the tumour microenvironment^56^. Our results support a specific role for NFATc3 in the positive *COX-2* regulation in U251 glioma cells, with clear therapeutic implication for the glioma growth. Interestingly, the opposite role of NFATc1 on *COX2* regulation will require to be further explored. The CXCL10/CXCR-3 axis has been shown to be expressed in glioma cells, and NFATc3 dependent regulation of CXCR3 is on line with the role of this receptor in migration and site specific dissemination in many cancerous cells^15,57–59^. Migration is a complex multistep process and we expect that many other factors, besides the genes explored in this study, contribute to cell migration. If these factors are NFATc3 dependent will need further analyses. Preliminary tests by using individual blocking antibodies against target genes were not sufficient to inhibit the migration of control glioma cells (data not shown).

Inhibition of NFATc-signalling using small molecule inhibitors is predicted to suppress tumorigenesis, hopefully without the secondary effects of the immunosuppressant regimes. Therefore, future cancer therapy targeting NFATc must take into account the different cellular types which participate in glioma microenvironment such as cancer cells, microglia and macrophages, and tools for specific delivery of this regulators will be required.

## Material and Methods

### Cell lines

*U87 and U251* human glioblastoma cell lines were purchased from ATCC and maintained in DMEM (LONZA), containing 1X Glutamine (LONZA), 10% Fetal Bovine Serum (FBS) (HiMedia), and 100 U/mL PenStrep (LONZA). Cells were genotyped using StemElite ID genotyping system (Promega).

*Human glioblastoma* (*hGB*) cell lines obtained from xenografts were obtained after injection of small pieces of human tumours (embedded in Matrigel, Corning) in the flank of immunodeficient mice, as described in^60^. Samples were obtained from patients treated at Hospital Universitario 12 de Octubre (H12O, Madrid, Spain). Informed consent from patients was obtained. None of them was under the age of 18th. The study was performed with the approval and following the guidelines of the Research Ethics Committee of Hospital 12 de Octubre (CEI 14/023).

#### U251-mCherry-Luc cells

U251 cells were transduced with lentiviral vector (mCherry-Luc) as below. After 48 hours, cultures were monitored for cherry fluorescence. Cherry positive cells were selected with 1 μg/mL puromycin in media and analysed by flow cytometry.

### Cell lysis and immunoblot analysis

For whole-cell extracts, cells grown and stimulated were washed twice with cold PBS and lysed for 30 min on ice in 100 μL hypertonic buffer as described^30^. Total extracts were resolved by 10% SDS-PAGE and proteins transferred to nitrocellulose membranes. The following antibodies were used: anti-NFATc1 [7A6, Alexis Biochemicals], NFATc3 [M75, Santa Cruz], NFATc2 [25A10.D6.D2, Abcam], NFATc4 [H-74, Santa Cruz] α-tubulin (Sigma), β-actin (Sigma), mouse anti RCAN1-4 (as described in^30^). Antibody was detected with enhanced chemiluminescence (ECL) detection reagent (GE Healthcare Lifesciences).

### RNA isolation and Real-Time PCR

Total RNA was extracted with Tripure (Roche), and 2 μg were reverse transcribed to cDNA with MMLV-RT (Invitrogen). Real-time quantitative PCR (qPCR) in duplicate was carried out on 100 ng cDNA, using TaqMan probes and SYBR Green system (Applied Biosystems) in ABI Prism 7900 or 7500 Fast Real Time PCR System (Applied Biosystems). Taqman probes used were NFATc1 (cat # Hs 00542678_m1), NFATc2 (cat # Hs 00234855_m1), NFATc3 (cat # Hs 00190046_m1), NFATc4 (cat # Hs 00190037_m1), COX2 (cat #Hs00153133_m1), IL2 (cat #Hs00174117_m1) RCAN1.4 (custom assay hDSCR1.4-E4_5 cat#4331348), Rcan1 (cat# Hs01120954_m1), IL8 (cat# Hs00174103_m1), VEGF (cat#Hs00173626_m1), TNFα (Hs00174128_m1). The following Taqman probes were used as endogenous controls: TFRC (cat #4333770 F), GAPDH (cat Hs99999905_m1) and 18 S rRNA (cat#4319413E). Relative quantification was calculated according to manufacturer’s instructions. Primers used for SYBR Green assays were as follow, TFRC was used as endogenous control.

CCL2: Forward: CAGCCAGATGCAATCAATGCC

Reverse: TGGAATCCTGAACCCACTTCT

CxCR3: Forward: TTTGACCGCTACCTGAACATAGT

Reverse: GGGAAGTTGTATTGGCAGTGG

GM CSF: Forward: GTGGCATTCAAGGAGTACCTC

Reverse: TGATGGCCTTCGATTCTGGATT

TFRC: Forward: GCAAGTAGATGGCGATAACAGT

Reverse: CAATAGCCCAAGTAGCCAATCAT

### Lentiviral vectors

Knockdown (KD) sequences were designed cloned into an EcoRI-NotI LV-Ubq-GFP lentiviral vector (LV) as described previously in^29^. The KD sequences employed were as in^29,61^.

KD-Sc (5′-3′): GATCCGGCAACAAGATGAAGAGCACCTCGAGTTGGTGCTCTTCATCTTGTTG

TTTTTGTTTAAACGC.

KD-c1 (5′-3′): GATCCCCGCCAGTACCAGCGTTTCACTTCAAGAGAGTGCGCTGGTACTGGCTTTTTGGAAC.

KD-c3 (5′-3′): GATCCCCGACAGTCGCTACTGCAAGCTTCAAGAGAGCTTGCAGTAGCGACTGTCTTTTTGGAAC

Lentiviruses expressing mCherry and luciferase genes (mCherry-Luc) were produced using PLKO-mcherry-luc-puro-Renilla cDNA (Addgene).

### Lentiviral production, titration and infection

Lentiviruses expressing mCherry-Luc and KD sequences were obtained by transient calcium phosphate transfection of HEK-293 cells with a three plasmid HIV-derived and VSV pseudotyped lentiviral system kindly provided by M. K. Collins (University College London, UK). Supernatant containing lentiviral particles was collected 48 h after removal of the calcium phosphate precipitate and was then ultracentrifuged for 2 h at 121,986xg at 4 °C (Ultraclear Tubes, SW28 rotor and Optima L-100 XP Ultracentrifuge, Beckman). Viruses were collected by adding cold sterile DMEM and were titrated by qPCR, as described by Scherr *et al*.^62^. U251 glioma cells were infected by adding virus (MOI = 6) and incubating for ON at 37 °C in complete medium DMEM 10% FBS and antibiotics. Infection efficiency (GFP or mCherry expression) and cell death (propidium iodide staining) were monitored by flow cytometry.

### Adenovirus Infection of U251 glioma cells

The adenovirus encoding constitutively active NFATc3 (Ad NFATc3 CA) and the control adenovirus (Ad Control) were as described^34^. Adenoviruses were generated and purified following the standard protocols^30^. Adenovirus infection was performed on subconfluent U251 glioma cells, and expression of the encoded protein was monitored by immunoblotting.

### Flow Citometry

Single cell suspensions were prepared in staining buffer (2% FBS) in PBS. Analysis was performed in a LRS Fortessa X-20 (BD Biosciences) cytometer, using the FlowJo v6.3.4 (TreeStar) and DIVA v8.0 software packages. Proliferation of infected U251 cells was analysed with the Violet Proliferation Dye 450 (BD Horizon). Infected cells were labelled according to the manufacturer’s protocol and stimulated with 2% FBS for three days. Violet staining was determined by flow cytometry in viable cells at day 0 and day 3 after stimulation. *Division Index*: the average number of cell divisions that a cell in the original population has undergone. *Proliferation Index*: the total number of divisions divided by the number of cells that went into division. Only takes into account the cells that underwent at least one division. Data was analysed with FlowJo 7.6.3 software (Tree Star, Inc.).

### Mouse brain xenograft assays

Housing and animal experiments were performed according to European Union 86/609/EEC guidelines and approved by the Animal Committee of Instituto de Salud Carlos III. All surgical procedures were carried out under general anaesthesia with 8 μL/g intraperitoneal (IP) anaesthetic solution composed of 10% ketamine (Imalgene) and 9.3% xylazine (Xilagesic) with subcutaneously administration of 10% analgesic solution (4 μL/g, Metacam). After surgery, mice were woken from anaesthesia with 7.7% reverting solution (4 μL/g, Antisedan). All of them prepared in PBS.

#### Orthotopic xenografts

gGuided intracranial injections using stereotactic frame (Leica) in athymic nude Foxn1nu mice (Harlan Iberica) were performed by administering 1 × 10^5^ of U251 transduced with lentiviral vector as indicated. Cells were resuspended in 2 μL PBS and injection were made into the striatum (coordinates: A-P, −0.5 mm; M-L, +2 mm; D-V, −3 mm; related to Bregma) using a Hamilton syringe. Animals were sacrificed at the onset of distress or when specify if symptoms were not apparent.

### *In vivo* imaging

Mice were injected with 100 μL of luciferin (12,5 mg/mL) in PBS (Becton Dickinson), anesthetized with isoflurane and placed in an IVIS Lumina XR Serie III (Caliper, BD), being kept with the same solution of 2% isoflurane gas in 100% oxygen at a flow rate of 0.5 mL/min during the process. Mice were placed in the IVIS and 5 images were taken every 30 seconds to get the signal peak. Imaging data are reported as (photons/sec/cm^2^/steradian).

### Tissue Processing

For confocal studies, mice were deeply anesthetized by intraperitoneal (ip) injection of a mixture of ketamine and xylazine and transcardially perfused with 25–30 mL of saline solution for 5 min, followed by 10 min with 4% PFA (Sigma), pH 7.4, in 0.1 M phosphate buffer (PB, Sigma). Brains were obtained and post fixed with 4% PFA for 18–20 h at 4 °C, rinsed in 0.1 M PB and placed in 15% glucose at 4 °C until they sank followed by immersion in 30% sucrose in PB at 4 °C for 72 h. Finally, brains were embedded in tissue freezing medium (Tissue-Tek O.C.T^TM^, Sakura), by submerging brains in increasing concentrations of OCT, snap frozen in dry-ice-cooled 2-methylbutane (Sigma) and stored at −80 °C. Coronal sections (30 μm) were obtained with a CM1950 cryostat (Leica Microsystems) and stored at −20 °C until use.

To perform qPCR on brain samples, brains were dissected and tumours were extracted and snap-freeze. Samples were liquid-nitrogen-cooled pestle and mortar fragmented for subsequent analysis.

### Confocal Microscopy and Analysis

Images were acquired on a Leica confocal microscope SP5 (Leica Microsystems). Brain maps were imaged using a 20X immersion objective and a 3.0 optic zoom (2.0 μm step size, 512 × 512 pixel resolutions). Map images are presented as maximum projections of z-stacks. Co-localization was measured on one z using the Leica LAS AF program to obtain Pearson coefficient.

### Migration assays

For wound healing assay, infected U251 cells were plated at 90% confluence in p35 dishes. After 24 h, monolayers were scratched with a p200 pipette tip. Cells were then washed and stimulated with medium containing 2% serum. Wound healing was monitored by time lapse microscopy with a NIKON Ti-Eclipse inverted microscope fitted with a 4X objective lens. Images were acquired with Nikon Nd2 Viewer and cell-free area was calculated with ImageJ software.

For transmigration assay, infected U251 cells (10 × 10^5^/well) were seeded onto 8 µm-pore transwell 24-well inserts (Costar) in DMEM 0.1% BSA (DMEM-BSA). 1 mL DMEM-BSA with or without 2% FBS was added in the lower chamber of the transwell, after 4 hours non migrating cells were removed and the filter was fixed in 4% paraformaldehyde and stained with Hoechst 33342 (Invitrogen). Migrated cells were counted with Image J software in four random fields in 4X pictures using a Nikon Eclipse TE2000-U Microscope in duplicates.

### Statistical analysis

Presented data is shown as means ± SD of three or more independent experiments. Statistical significance was estimated by Student’s t using GraphPad Prism 5.0 (GraphPad Software). Differences were considered significant at *p < 0.05. For immunoblots, a linear correlation was observed between increasing amounts of input protein and signal intensity.

### Database analysis

TCGA expression data (http://cancergenome.nih.gov/) were analysed using GlioVis (http://gliovis.bioinfo.cnio.es/) data portal for visualization and analysis of brain tumour expression datasets^63^. Data downloaded were analysed using GraphPad Prims 5.0.

## Supplementary information

Suplemental material
