# Supplementary material for: NFATc3 controls tumour growth by regulating proliferation and migration of human astroglioma cells
Source: Sci Rep. 2019 Jun 27;9:9361. doi: 10.1038/s41598-019-45731-w (PMC6597574; doi:10.1038/s41598-019-45731-w)
Supplement: Supplementary file 1 — Suplemental material [file 41598_2019_45731_MOESM1_ESM.pdf]

NFATc3 controls tumour growth by regulating proliferation and migration of human astroglioma cells

Katia Urso[4#], Andrés Fernández[1#*], Patricia Velasco[1], Javier Cotrina[1], Belén de Andrés[2], Pilar Sánchez-Gómez[1], Aurelio Hernández-Laín[5], Sonsoles Hortelano[3], Juan Miguel Redondo[4] and Eva Cano[1*]

Supplemental Figure S1

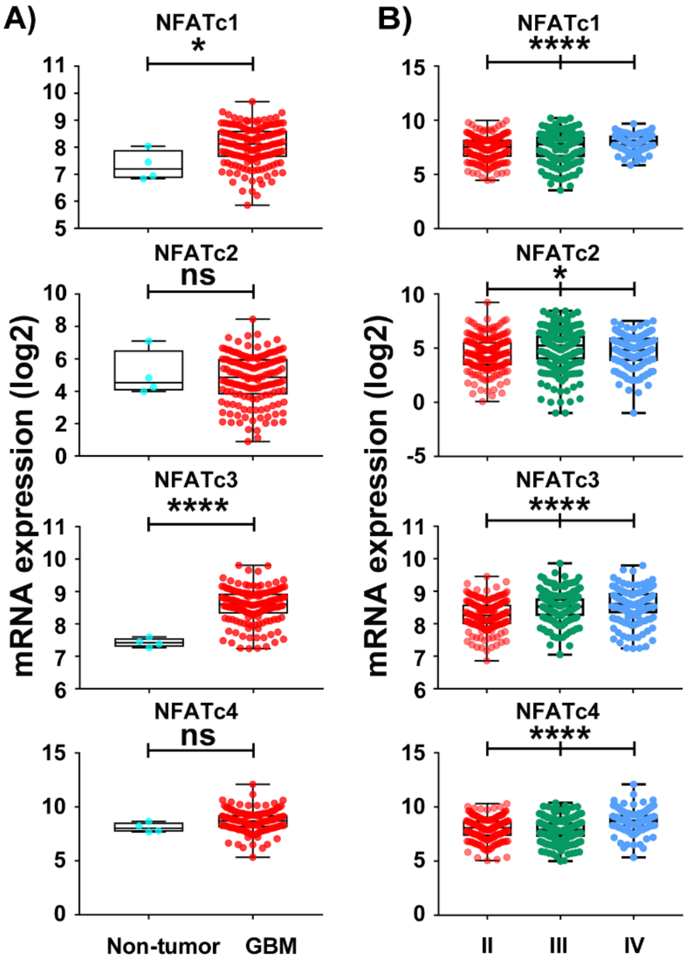

Supplemental Figure S1: Analysis of NFATc family member's expression using TCGA databases. Interrogating RNAseq in TCGA studies (http://cancergenome.nih.gov/) using Gliovis tools (http://gliovis.bioinfo.cnio.es/). Data were downloaded and analysed using GraphPad software. Significance results are indicated. A) Comparison of normal and tumour samples using TCGA_GBM study. 160 cases, 4 non-tumour and 156 GBM. . c1: *$P < 0.05$, c2: $^{ns}P > 0.05$, c3: ****$P < 0.0001$, c4: $^{ns}P > 0.05$ (t-test). B) Expression and tumour progression across glioblastoma tumour grade II, III and IV using TCGA_LGG data. 620 cases. c1: ****$P < 0.0001$, c2 *$P < 0.05$, c3: ****$P < 0.0001$, c4: ****$P < 0.0001$ (ANOVA).

Supplemental Figure S2

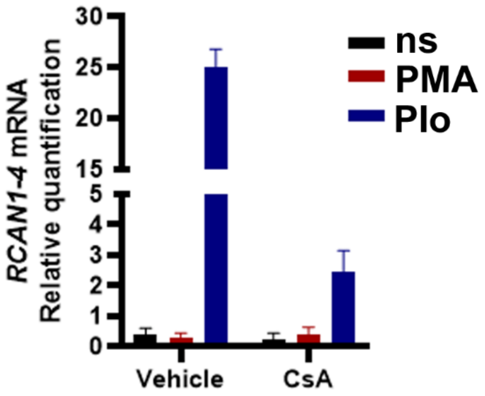

Supplemental Figure S2. PMA alone is not sufficient to promote NFAT-dependent *RCAN1-4* expression. U251cells were pre-treated without or with CsA (200 ng/mL) and then stimulated for 4 hours with PMA (20 ng/ml) or in combination with calcium ionophore, Io (1μM), PIo as indicated, RCAN1-4 mRNA was amplified from total RNA by TaqMan RT-PCR. RCAN1-4 mRNA was quantified in arbitrary units normalized to the expression of human TBP. Levels are presented as the fold expression above non stimulated, ns, cells. Values are means ± SD of RT-PCR determinations for each condition.
